# Feasibility and safety of biologic OviTex mesh in ventral mesh rectopexy: a prospective pilot study

**DOI:** 10.1007/s10151-024-03097-w

**Published:** 2025-02-13

**Authors:** M. A. Boom, E. M. van der Schans, N. A. T. Wijffels, P. M. Verheijen, E. C. J. Consten

**Affiliations:** 1 Department of Surgery, Meander Medical Centre, Amersfoort, The Netherlands; 2 Department of Surgery, UMC Utrecht, Utrecht, The Netherlands; 3 Department of Surgery, UMC Groningen, Groningen Amersfoort, The Netherlands; 4 Department of Surgery, St. Antonius Hospital, Nieuwegein, The Netherlands; 5 Faculty of Electrical Engineering, Mathematics and Computer Science, Institute of Technical Medicine, Twente University, Enschede, The Netherlands

**Keywords:** Rectal prolapse, Ovitex, Biological mesh, VMR, Feasibility study, Pilot

## Abstract

**Background:**

Minimal-invasive ventral mesh rectopexy (VMR) is a widely accepted treatment for patients suffering from rectal prolapse. The type of mesh used in VMR remains a subject of debate. Currently, the most applied implant is a polypropylene mesh. The aim of the present pilot study was to determine the ease of use, feasibility, and safety of OviTex PGA mesh, a biologic mesh, in VMR.

**Methods:**

Consecutive patients who underwent VMR for internal or external rectal prolapse were included in a prospective non-randomised pilot study in two centers. Preoperative and postoperative evaluation (90 days and 6 months) with a clinical examination and questionnaire regarding pelvic floor symptoms was performed. The primary objectives were to monitor the perioperative technical end result and the postoperative complication rate.

**Results:**

Sixteen patients underwent VMR with an OviTex PGA implant. All operations were completed successfully and without intraoperative complications. The mean ODS and FISI score was significantly decreased after 6-months follow-up. No graft-related complications (GRC) occurred. Two patients developed a recurrent prolapse within 6 months.

**Conclusion:**

Robotic correction of rectal prolapse using an OviTex mesh is a safe, minimally invasive, technically feasible treatment. However, further research is warranted to evaluate the potential added value of OviTex compared to polypropylene mesh on a larger scale. Long-term follow-up is essential to assess the durability of the procedure and monitor the occurrence of any new symptoms.

## Introduction

Minimal-invasive ventral mesh rectopexy (VMR) is a widely accepted treatment for patients suffering from rectal prolapse [1–3]. Choice of material used in VMR—synthetic or biologic surgical mesh—remains subject of debate. The discontinuation of transvaginal mesh sales and distribution by the FDA in April 2019, prompted by complications, has unjustly impacted the perception of surgical mesh for ventral mesh rectopexy (VMR), despite the comparatively lower rates of erosion and complications associated with VMR mesh. Currently, the most widely used mesh in VMR is polypropylene, and this has shown good results regarding recurrence, mesh exposure, and functional outcome [4–6]. Although complication rates are low, the serious complications of fistulation and mesh exposure are reasons to opt for a more expensive biological mesh. Over the past few years, biologic meshes have been studied as possible alternatives to synthetic materials. These meshes potentially offer advantages, including a decreased incidence of fistulae, a milder immune response, and reduced fibrosis [4, 7, 8]. Therefore, it is not surprising that interest in biological meshes for surgical repairs, such as VMR, is increasing. However, there are some concerns regarding long-term mechanical strength, which could lead to more recurrences. The available literature does not suggest a different performance of biologic mesh over synthetic mesh regarding either risk of recurrence or exposure. However, only limited data on biologic mesh VMR are available [9].

Thus far, the two types of biologic mesh studied in VMR are Biodesign and Permacol. A novelty on the mesh market is OviTex. It is a non-crosslinked biologic mesh fabricated from sterile ovine (sheep) extracellular matrix (ECM). The uniqueness of OviTex lies in its composition, which consists of essential components necessary for tissue regeneration. It encompasses a diverse array of matrisome proteins, including collagens, glycoproteins, proteoglycans, ECM-affiliated proteins, ECM regulators, and secreted factors. This comprehensive combination of proteins contributes to OviTex's remarkable ability to mimic the native extracellular matrix, making it a highly promising biomaterial for successful tissue repair and regeneration [10]. Various material configurations have been developed and are currently accessible in the market, featuring multiple layers of biologic materials reinforced by either permanent (polypropylene) or resorbable (polyglycolic acid) polymer stitching. The combination of the biological healing support of an ovine-derived matrix with the structural benefits of synthetic reinforcement makes it versatile and cost-effective for diverse surgical needs. In contrast, Biodesign and Permacol focus on purely biological solutions, with differences in source material and processing that influence their specific applications and performance in tissue repair and regeneration. Based on these features, data from animal studies, and the first prospective and retrospective studies in humans, using OviTex may lead to better outcomes than other biologic meshes used in VMR [11–13].

To date, OviTex has only been studied in abdominal hernia repairs [10]. With no reports on OviTex in VMR, a pilot study should be conducted first to test the feasibility and safety in VMR as a basis for future larger studies. The aim of the present pilot study was to determine the ease of use, feasibility, and safety of OviTex mesh in VMR.

## Methods

### Study design and patients

This study was performed as an observational single-arm pilot study in two centers in order to test OviTex PGA (polyglycolic acid), a resorbable synthetic grid, as a novel therapy for patients with rectal prolapse. All consecutive patients scheduled for VMR were asked to participate and were included after completion of an informed consent process. Indication for surgery was full thickness rectal prolapse, external rectal prolapse (ERP), or an Oxford grade III/IV symptomatic internal rectal prolapse (IRP) failing to respond to conservative therapy. The diagnosis of rectal prolapse was made clinically and in case of IRP confirmed by dynamic pelvic MRI. Subjects were eligible if they were > 18 years old. Exclusion criteria were previous rectal prolapse surgery, an allergy to ovine rumen, mental incompetence that prohibited completion of questionnaires, a history of pelvic radiation therapy, and deviation from the surgical protocol. In addition, patients with concurrent anterior or middle compartment abnormalities requiring additional surgery to VMR were not eligible. Sample size was not calculated because this pilot study aimed to provide a descriptive evaluation of the safety and feasibility of the OviTex in VMR. Thus, 15 participants were considered appropriate.

The study protocol was reviewed and approved by the respective Institutional Review Boards of both centers (NL74593.100.20).

### Surgical procedure and materials

**Fig. 1:**
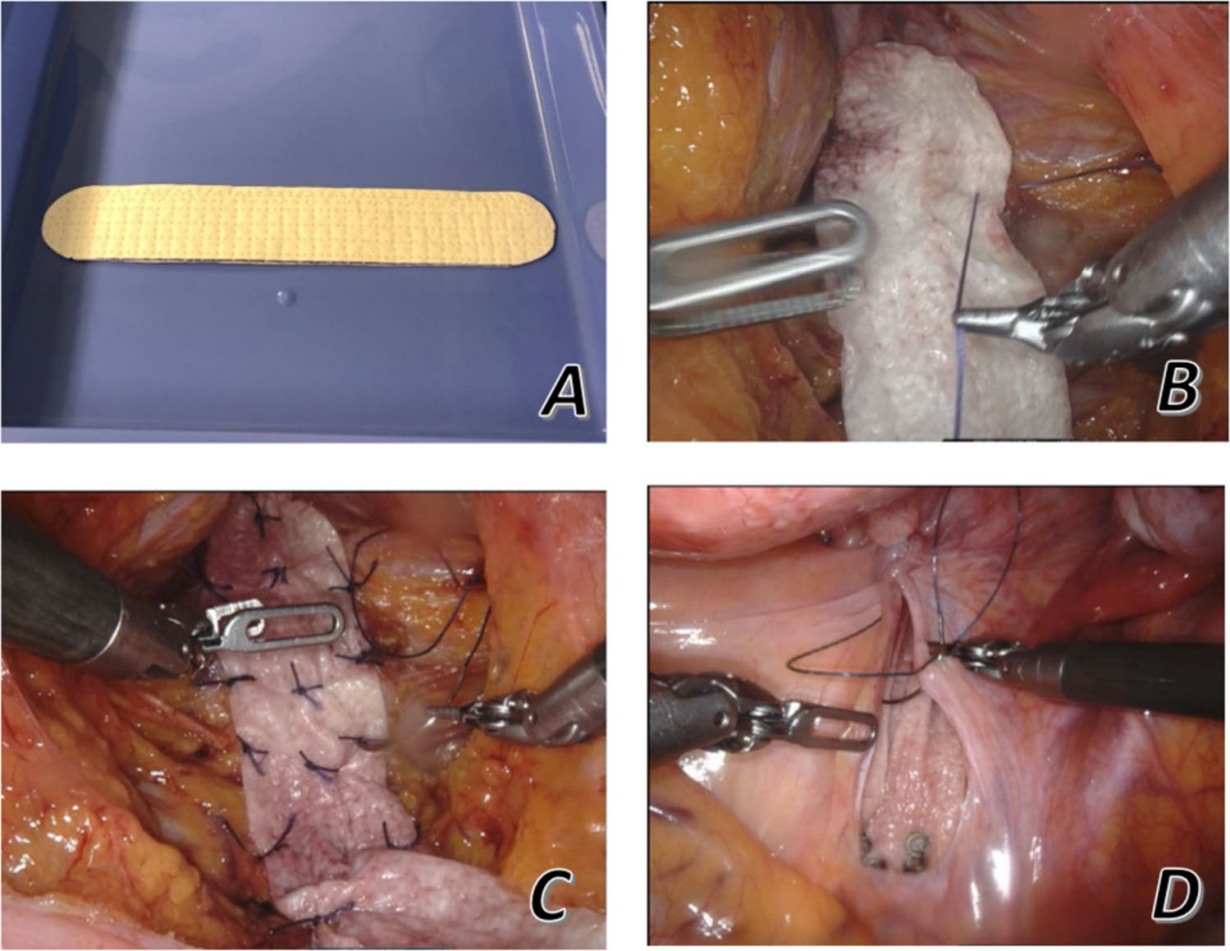
Stages in the ventral rectopexy process involving the placement and attachment of biological mesh. OviTex Core PGA graft tailored and hydrated (**A**), fixation of the prosthesis distally (**B** and **C**), proximal end fixation with tacks and buried in the peritoneum (**D**)

In both institutions, minimally invasive surgery was performed. Patients underwent robotic ventral mesh rectopexy (RVMR) utilizing the Da Vinci robotic system by Intuitive Surgical. All procedures were performed by three experienced surgeons who have extensive experience with VMR. The operative technique is similar to the originally described procedure by D’Hoore and Penninckx and has been described in detail previously [1, 14]. The only distinction from the originally described technique was using a 3 × 15-cm OviTex Core PGA graft instead of a synthetic mesh. The OviTex Core PGA was tailored, hydrated, and introduced to the abdominal cavity through the assistant port (Fig. 1).

The selection of OviTex Core PGA mesh in this study was driven by its distinctive characteristics as a completely resorbable variant, providing the benefit of the lowest synthetic load compared to other OviTex variants that utilize the polypropylene grid. This resorbable nature of OviTex PGA mesh reduces the long-term presence of synthetic material, potentially minimizing the risk of foreign body response and facilitating natural tissue healing and remodeling processes. Furthermore, OviTex offers a cost-effective solution relative to other biological meshes. The mesh is fixed to the ventral aspect of the distal rectum using multiple sutures. OviTex was sutured to the ventral aspect of the distal rectum with vicryl 2–0 in the first eight patients. In response to an early recurrence, a modification was made to the surgical procedure. Material for distal mesh fixation was changed during the study from only vicryl 2.0 to both vicryl 2.0 and PDS 3.0 suture (Ethicon, Johnson & Johnson, Hamburg, Germany). This decision was influenced by the distinctive resorption characteristics of these materials. Vicryl, known for its higher tensile strength, is recognized for its relatively rapid decrease in tensile strength within a brief period, typically occurring over 3–5 weeks. In contrast, PDS 3.0 demonstrates a notably extended resorption timeframe, ranging from 60 to 210 days. The underlying rationale was that, by the time PDS 3.0 had completely resorbed, a sufficient quantity of collagen should have been synthesized within the patient's tissues, typically occurring after a 90-day complete resorption of OviTex core PGA. On the promontory, the proximal end of the mesh is fixed utilizing titanium tacks (Autosure Protack 5 mm; Covidien, USA). Lastly, the peritoneum is closed over the mesh with a 23-cm V-lock suture (Covidien,Mansfield, MA, USA) until the mesh is completely covered with peritoneum.

### Outcome measures

This pilot study examines the safety and feasibility of using OviTex core PGA during VMR. Feasibility was assessed by a questionnaire rating the perioperative technical end result with OviTex via three five-point Likert scale statements. This scale ranges from much worse, worse, equal, better, and much better in use compared to their previous experience with polypropylene regarding: (1) insertion of the mesh into the abdominal cavity, (2) fixating the mesh to the rectal wall, and (3) fixating the mesh to the promontory. The questionnaire also assesses possible perioperative complications and asks surgeons to rate their overall experience regarding the technical end result.

Safety was assessed by monitoring complications in the 90-day postoperative period. This included physical examination and rectoscopy to assess graft-related complications (GRC), including mesh exposure. Peri- and postoperative complications were classified using the Clavien-Dindo classification [15]. Grades 1 and 2 were classified as minor and grade ≥ 3 as major complications.

Secondary endpoints were functional outcomes. For symptom evaluation, all patients completed a standardized questionnaire before and 6 months after surgery. The questionnaire included scores for obstructed defecation syndrome (Altomare) and fecal incontinence [fecal incontinence severity index (FISI)]. Additionally, patients were asked to rate their overall satisfaction using the patient global improvement (PGI-I) scale at 6 months.

### Statistical analysis

Completed responses were entered into the data management software program Castor EDC. The data were analyzed using SPSS (version 24). Patient characteristics and functional data were presented as mean ± standard deviation. Non-normally distributed data were reported as the median along with the interquartile range (IQR). The Wilcoxon signed rank test was used for non-parametric paired data and a t-test for paired and unpaired samples. Postoperative complications were compared using a chi-square test. A *p*-value < 0.05 was considered statistically significant.

## Results

### Demographics and characteristics of participants

In total, 16 patients were treated with OviTex PGA VMR from January 2021 to November 2021. A summary of patient demographics and characteristics is given in Table 1. The mean age was 52.7 (SD 21.3) years. Indication for surgery was ERP in eight (50%) patients and IRP in eight (50%) patients. One (6.3%) IRP patient had a concomitant enterocele. Regarding defecatory problems, patients had complaints of fecal incontinence (43.7%), obstructed defecation (43.8%), or both (12.5%). Missing items in the questionnaires ranged between 1–8% (Table 2).

**Table 1:** Baseline characteristics of patients

Baseline characteristics	Total *N* = 16
Female, *N* (%)	14 (77.8)
Age (years), mean ± SD	53.4 ± 21.2
BMI (kg/m³), median [IQR]	22.48 [6.22]
ASA, *N* (%)	
1	5 (31.3)
2	10 (62.5)
3	1 (6.3)
4	–
Type of prolapse, *N* (%)	
ERP	8 (50)
IRP	8 (50)
Rectocele	8 (50)
Enterocele	1 (6.3)
Complaints, *N* (%)	
ODS	10 (62.5)
FI	6 (37.5)
Bulge feeling	2 (12.5)
Urinary incontinence	2 (12.5)
Surgical history, *N* (%)	
Hysterectomy, vaginal	2 (12.5)
Hysterectomy, abdominal	–
Anterior colporrhaphy	1 (6.3)
Posterior colporrhaphy	2 (12.5)
Sacrospinal fixation (no mesh)	1 (6.3)
PPH for mucosal prolapse	–
Hemorrhoidectomy/RBL	1 (6.3)
Mucopexy	1 (6.3)
Other abdominal surgeries	4 (25)
No abdominal/pelvic surgery	6 (37.5)

*ASA* American Society of Anesthesiologists, *BMI* body mass index, *ERP/IRP* external/internal rectal prolapse, *IQR* interquartile range, *N* number, *PPH* procedure for prolapse and haemorrhoids, *RBL* rubber band ligation, *SD* standard deviation, *VMR* ventral mesh rectopexy

**Table 2:** Complications and recurrences

	OviTex (*n* = 16)
Complication^a^	
No complications	15/16
Minor	1/16
Major	0/16
Mesh-related complications	0/16
Recurrence	2/16

^a^Clavien-Dindo classification. Grades 1 and 2 were classified as minor and grade ≥ 3 as major

### Feasibility & safety

**Fig. 2:**
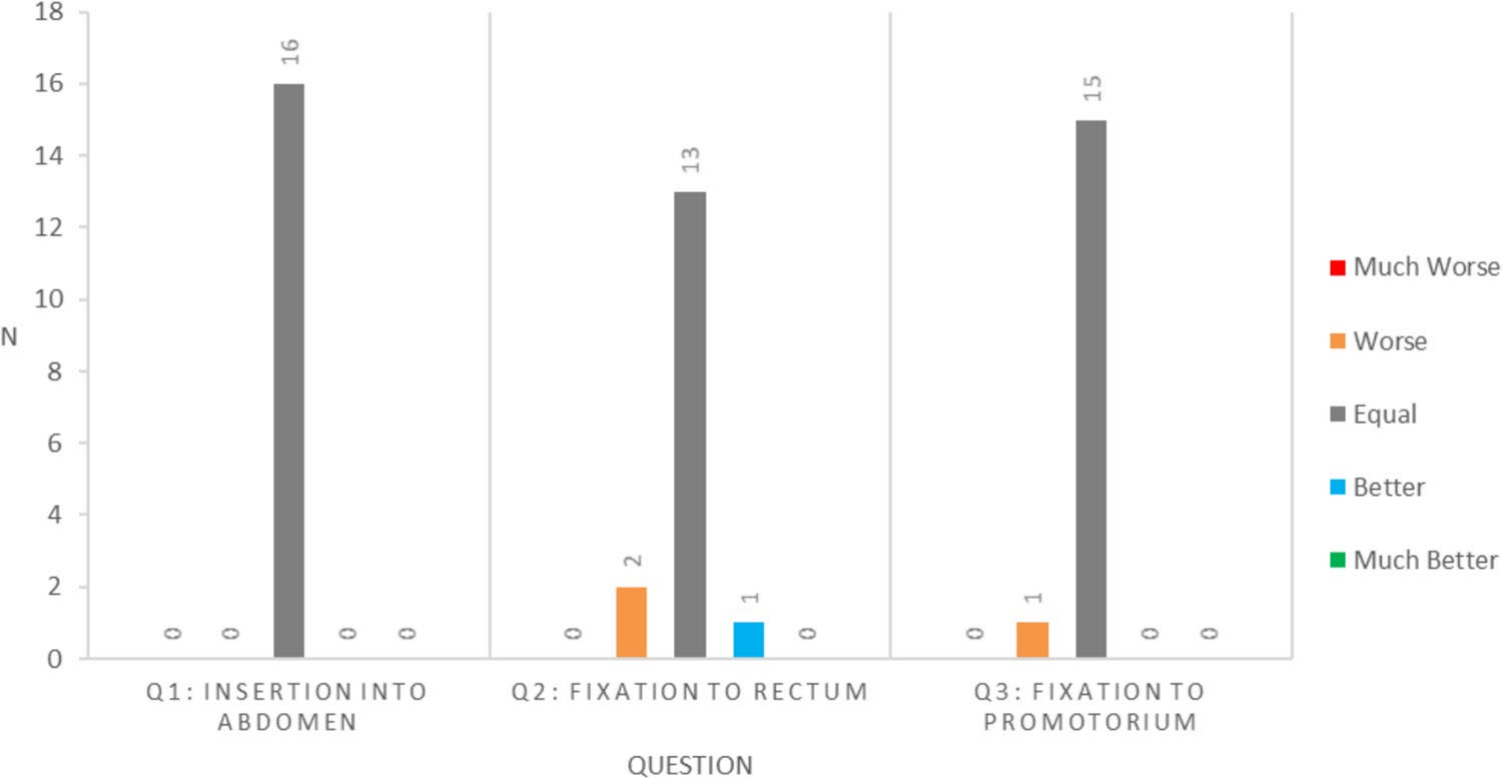
Summery of responses postoperative five-point likert scale

Technical results were scored postoperatively by the performing surgeon for all participants. Results are illustrated in Fig. 2. All reported ‘no difference’ regarding insertion of the mesh into the abdomen. In two cases, fixation to the rectum was reported as more difficult and in one case as easier. Fixation to the promontory was reported as more difficult in one patient. None of the surgeons reported ‘far easier’ or ‘way more difficult’ in any of the three statements.

Complications occurred in 1 out of 16 patients. This minor complication concerned an oral fungal infection possibly due to prolonged intubation. No GRC and mesh exposure occurred.

### Recurrence

Two patients presented with a recurrent rectal prolapse at 90 days, both in patients with full-thickness rectal prolapse. No cause could be identified. The first patient, a 19-year-old woman, had had a rectal prolapse since infancy. Six months after the prolapse recurrence, she underwent a resection rectopexy. This operation was successful. The second patient, an 18-year-old male, presented after 90 days with an external rectal prolapse. Considering the mild functional complaints, the decision was made to first resume intensive physiotherapy.

### Functional outcomes

All of the 16 enrolled subjects completed the 6-month follow-up. One subject was not able to fill in the postoperative questionnaire due to vascular dementia which was diagnosed after inclusion. FISI score was significantly reduced at 6 months (pre- vs postoperatively: 45.40 ± 12.712 vs 38.40 ± 12.235 *P* < 0.05) (Table 3). ODS score declined from the preoperative value of 14.75 (± 5.939) to 8.93 (± 4.200) postoperatively (*P* < 0.01). No severe constipation or new-onset defecatory complaints were observed.

**Table 3:** Pre- and postoperative FISI and ODS

	Preoperative (*n* = 16)	Postoperative (*n* = 15)	Significance (paired test)
FISI	45.40 ± 12.712	38.40 ± 12.235	*P* < 0.05
ODS (Altomare)	14.75 ± 5.939	8.93 ± 4.200	*P* < 0.01

Postoperative functional outcomes were assessed at the 6-month mark

Data are presented as the mean ± standard deviation. Statistical signifcance was set at *P* < 0.05

### Patient satisfaction

Ten of 16 patients described their condition as being (much) improved after VMR with OviTex. Two (*n* = 2) reported they were ‘about the same’ and another two reported that their health status was worse compared to their preoperative status.

## Discussion

Ventral mesh rectopexy has emerged as the preferred procedure for treating patients with rectal prolapse. With the risk of serious graft-related complications and increased negatively influenced perception of synthetic surgical mesh, the need for an alternative treatment with biologic mesh is growing. Lately, the first studies investigating biological grafts (i.e., Biodesign and Permacol) have been published, showing promising results [16–19]. OviTex is a biologic mesh thought to have similar characteristics but is available at a lower cost, which makes it an attractive alternative. This pilot study is the first study to investigate an OviTex mesh used in VMR.

In the present study, we aimed to determine the feasibility and safety of the OviTex mesh in VMR. The perioperative technical results with OviTex were found to be comparable to polypropylene in 91.6%. In the initial cases, the fixation to the rectum and to the promotorium was assessed as slightly more difficult. This was related to the non-transparent nature of the mesh. During the study, surgeons adjusted to this quickly.

During the follow-up period, no GRC occurred. The risk of GRC increases with time. A review and meta-analyses describe a GRC rate of 0–2.4% [9], with time to event ranging between 2 to 78 months. However, the possibility of missed vaginal erosion cannot be excluded as the follow-up was limited to rectoscopy.

The aim of surgery is to correct anatomical alterations and mitigate symptoms. The mesh provides a scaffold keeping the rectum in place until new patient-derived healthy collagen tissue has replaced the graft. Published recurrence rates after VMR range from 1.1% to 18.8% [9, 20, 21]. For biologic meshes, incidences range from 0 to 15.4% [7, 9, 22]. In our study, 2 (12.5%) out of 16 patients who completed 6-month follow-up showed an anatomical recurrence. This should be interpreted with caution as this study was not powered to evaluate recurrence rates and follow-up was limited. The initial recurrence observed in our study involved a patient who underwent a suture rectopexy after 6 months, where the remaining matrix and collagen deposition remained visible proximally but not distally. This disparity suggests insufficient tissue remodulation or a potential detachment of the mesh in that region. It is possible that early recurrences are secondary to technical failures though a mesh failure or insufficient tissue remodeling cannot be excluded. Since the first recurrence occurred in the third patient, material for distal fixation was reviewed halfway through the study period, considering suture material choice may contribute to failure. Nevertheless, the second recurrence occurred in the eighth patient in which both vicryl and PDS sutures had been used. Both patients had had prolonged straining associated with constipation since infancy. Despite the attempts to prevent straining and constipation after prolapse surgery, it is possible that the habit persisted and provoked early recurrence. However, we do not have the data to support this.

Although the causes could not be identified, both recurrences appeared at around 90 days. By this time, the OviTex PGA implants have been resorbed, and sufficient collagen should have been formed by the patient [11]. In these patients, it may be that tissue remodeling was still in progress while the mesh lost its temporary strength. Based on these findings, it can be argued that there is a rationale for considering the use of a permanent synthetic fiber to enhance the durability of prolapse repair and reduce the likelihood of recurrences. It is important to note that within the range of OviTex mesh products, various variants are available, including one with a permanent grid structure of polypropylene that may offer extended longevity. Although resistance against synthetic grafts is growing, ‘OviTex Permanent’ contains 96% less polymer than the standard polypropylene mesh. Furthermore, the polymer is embedded in the ECM, which is thought to attenuate any inflammatory response [23]. Observations in primates show that a minimized amount of embedded synthetic reinforcement results in an implant that histologically behaves like a biologic mesh yet maintains its functional structure [11].

Regarding patient satisfaction, two patients reported a worse state of health compared to baseline. For the first patient, indication for surgery was IRP and rectocele combined with functional complaints of fecal incontinence. No improvement in symptoms was seen during follow-up, although postoperative dynamic MRI showed absence of IRP or rectocele and thus a correctly restored anatomy. This lack of symptom improvement despite correctly restored anatomy aligns with earlier studies that showed an improvement in fecal incontinence rates for IRP patients in 69% to 76.5% of patients [17, 24, 25]. The second patient had preoperative complaints of pain, in addition to anatomical and functional abnormalities, which persisted after the operation against patient expectations. Although the functional symptoms were resolved, the persistent pain may have contributed to lower satisfaction. This aligns with a study by Singh et al., where 32% of patients reported postoperative pelvic pain (PP) at follow-up, with 65% having it preoperatively. Dissatisfaction with VMR outcomes was noted in 41% of patients experiencing PP at follow-up, emphasizing the impact of pre-existing conditions on postoperative satisfaction [24].

Due to the pilot study design, there are several limitations. First, a small number of patients was included, and follow-up was short. Second, a relatively large number of ERP’s was included in this report (50%). Notably, ERP is associated with substantially higher recurrence rates [2, 26]. However, this difference may partly reflect the fact that anatomical recurrences in ERP are more readily diagnosed clinically compared to IRP.

In conclusion, our results demonstrate the feasibility and safety of utilizing OviTex in VMR. However, the presence of two early recurrences raises questions about the suitability of OviTex PGA for certain cases. In our opinion, the next appropriate step would be to conduct a pilot study with OviTex with a permanent grid to further investigate its potential benefits. Based on these results, future studies should involve a larger cohort of patients to further investigate the efficacy of OviTex with a permanent grid. Particularly intriguing would be the execution of a prospective and comparative study utilizing a randomized controlled design comparing OviTex with the conventional standard polypropylene mesh. Long-term follow-up is crucial to ascertain the durability of repairs and monitor the outcomes.

## Data Availability

No datasets were generated or analysed during the current study.

## Abbreviations

VMR: Ventral mesh rectopexy
GRC: Graft-related complications
ECM: Extracellular matrix
PGA: Polyglycolic acid
RVMR: Robotic ventral mesh rectopexy
ERP: External rectal prolapse
IRP: Internal rectal prolapse
